# Microcomputed Tomography and Histological Study of Bone Regeneration Using Tooth Biomaterial with BMP-2 in Rabbit Calvarial Defects

**DOI:** 10.1155/2021/6690221

**Published:** 2021-05-10

**Authors:** Puneet Wadhwa, Jeong Hun Lee, Bing Cheng Zhao, HongXin Cai, Jae-Suk Rim, Hyon-Seok Jang, Eui-Seok Lee

**Affiliations:** ^1^Department of Oral and Maxillofacial Surgery, Graduate School of Clinical Dentistry, Korea University Guro Hospital, Seoul 08308, Republic of Korea; ^2^The CONVERSATIONALIST Club, School of Stomatology, Shandong First Medical University & Shandong Academy of Medical Sciences, Tai'an, Shandong271016, China

## Abstract

Our study was aimed to analyze the osteoinductive effect of powdered and block type autogenous bone graft along with bone morphogenetic protein (BMP-2) as compared to synthetic bone graft. Three circular bicortical defects were made in the calvaria of each rabbit and randomly divided into three groups as follows: powdered tooth biomaterial+BMP-2, block tooth biomaterial+BMP-2, and control group: synthetic bone+BMP-2. The samples taken from these defects after 4 and 8 weeks were analyzed histologically along with micro CT analysis. In our study, both powered and block type tooth autogenous bone graft successfully stimulated mesenchymal cells leading to endochondral ossification and bone regeneration. We observed that the powered bone graft material which is acid insoluble especially is preferable as a carrier for BMP-2.

## 1. Introduction

Bone regeneration in the oral and maxillofacial region allows for the recovery of the esthetics and function of the craniofacial skeleton. The size of bone particles used for augmentation was reported to alter results in animal studies. Bone graft materials with an adequate particle size are essential, as autogenous bone grafts with larger particle size exhibited superior bone augmentation than grafts with smaller particle size [1]. In 1994, Mowlem suggested that smaller-sized bone particles augment the osteogenic effect of an autogenous bone graft [2]. Small particle grafts presented the satisfactory formation of bone trabeculae. Robinson hypothesized prompt resorption and replacement of small particles in the osseous coagulum technique as compared to larger particles [3]. Also, smaller fragments appeared to reinforce osteogenesis. Recently, the use of human dentin from extracted teeth as autogenous bone grafts has been quite intriguing for researchers and clinicians [4]. The autografts are the gold standard for bone repairing grafts because of their property for stimulating new bone growth [5]. The bone formation potential of dentin osteoinductivity was also observed in several studies. The dentin consists of 70-75% inorganic and 20% of organic constituents, like that of alveolar bone (inorganic: 65% and organic: 25%). The inorganic content mainly consists of hydroxyapatite and the main organic content of dentin is type 1 collagen, which contributes in bone regeneration and mineralization. Bone morphogenetic proteins (BMPs) are also found in the inorganic components of dentin. BMP helps in the differentiation of mesenchymal stem cells into chondrocytes which further contributes to bone formation. BMP derived from the teeth of various animals have shown osteoinductive properties and play an important role in bone formation size [6–8]. However, the amount of BMP extracted from a tooth is limited, hindering its clinical usage. This shortcoming can be overcome by gene recombination methods by preparing recombinant human BMP-2 (rhBMP-2) proteins based on mammal cells or colon bacilli. Thus, demineralized dentin matrix (DDM) may play the role of a potential scaffold material for releasing BMPs [9]. Despite the low amount of BMP in a debilitated tooth, active new bone formation was detected with DDM used as carrier size [9–11]. DDM scaffold distributes inductive cells to the healing site which gives cues for regulating the architecture of the tissue being formed [12]. Similarly, its osteogenesis is affected by the size and form of the biomaterial.

The objective of this study was to investigate the increase in osteoinductive activity due to tooth biomaterial different shapes with rhBMP-2 in a rabbit calvarial defect compared to a synthetic bone graft by histological and microcomputed tomography (CT) analysis at 8 weeks.

## 2. Materials and Methods

This study was performed with approval from the Institutional Animal Care & Use Committee of Korea University (KUIACUC-2013-168). Twelve healthy New Zealand white rabbits weighing approximately 2.5 kg each were used. Each animal was kept in a separate cage at our animal research lab. The animals were given enough water and diet throughout the experiment under 12 hour light and dark cycle. The animals were well observed.

The extracted rabbit tooth was submerged in 70% ethyl alcohol after which it was pulverized into different sizes using auto tooth biomaterial (Korea Tooth Bank, Seoul, Korea). Debridement of the tooth surface to remove soft tissue, calculus, or foreign substance attached to the surface was done. The crown and root were then separated and crushed.

Small particle size was in the range of 100 to 300 *μ*m, while block particles ranged from 1000 to 2000 *μ*m (standardized by sieves). The tooth particles were added to a mixture of distilled water and hydrogen oxide, and then, residual foreign substances were removed by washing with an ultrasonography washer. Then, an ethyl alcohol solution is used for dehydrating the tooth particles and ethyl ether for defatting. Sterilization of tooth allograft material is done with ethylene oxide gas and sealed.

A total of thirty-six calvarial defects (8-mm diameter) were randomly divided into 3 experimental groups as follows: group 1 (*n* = 12): powder-type graft+BMP-2; group 2 (*n* = 12): block-type graft+BMP-2; group 3, control group (*n* = 12): synthetic bone (Hydroxyapatite, Ca10[PO4]6[OH]2, Bongros, Bio Co., Korea)+BMP-2. BMP-2 used in this study was 0.5 mg rhBMP-2 Novosis Dent Daewoong Pharmaceuticals Co. Korea.

### 2.1. Surgical Procedure

**Figure 1:**
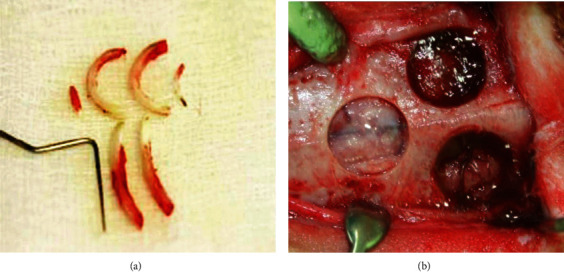
(a) Extracted rabbit teeth that were submitted to Korea tooth bank. (b) Demineralized dentin matrix with different particle sizes.

All surgical procedures were performed under general ketamine HCl (30 mL/kg) and xylazine HCl (10 mg/kg) and local (2% lidocaine with 1 : 100,000 epinephrine) anesthesia. The head of the rabbit was shaved, and povidone iodide solution was used for disinfection of the surgical site. After anesthetic and disinfection, incision, flap reflection, and exposure of the rabbit cranium were performed. Three bone defects (8-mm diameter) were prepared with a trephine bur in each animal (Figure 1). The bone defects were randomly filled with the experimental materials. Periosteal flap was closed using a 4-0 vicryl (Ethicon, Auneau, France) resorbable suture with simple interrupted stitches, and 4-0 nylon (Polyamide, AILEE CO. Busan, Korea) suture was used for closing the skin. Animals were sacrificed after 4 and 8 weeks.

### 2.2. Micro-CT Analysis

**Figure 2:**
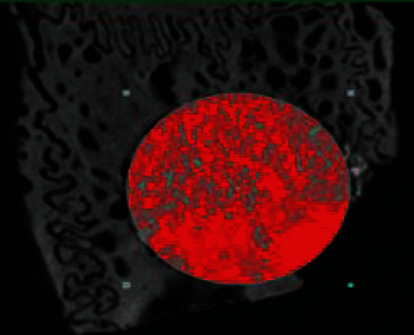
Measurement of calvarial sample under micro-CT (tissue volume, bone volume, percent volume) SkyScan 1173 (High-resolution micro-CT scanner SkyScan, Aartselaar, Belgium).

SkyScan 1172 high-resolution X-ray micro-CT system (Bruker Micro CT, Kontich, Belgium) operating at 80 kV and 124 *μ*A was used for scanning the samples. In order to generate high definition radiographic projection images, the experimental conditions were optimized accordingly. The region of interest was selected around the bone grafting area which corresponded with the defect size of 8 mm (Figure 2). These digital images were then refined for obtaining reconstructed cross-sectional images with the help of a mathematical algorithm. This algorithm was based on a filtered back-projection procedure implemented in the SkyScan NRecon Software (Bruker, Kontich, Belgium).

### 2.3. Histological Analysis

After micro CT analysis, the calvarial specimens were decalcified in formic acid. The specimens were trimmed and cut across to obtain a slice from the central part of the healing bone defect and embedded in paraffin. Serial sections (4-*μ*m) were cut from each part and stained with hematoxylin and eosin. The sections were perpendicular to the sagittal suture to form a plane of analysis passing through the center of the defect. Three central sections were examined for histological evaluation. The slides were examined under a light microscope to examine bone regeneration qualitatively. All sections were photographed.

### 2.4. Statistical Analysis

The data were analyzed with the SPSS version 20 software (SPSS, Inc.). The Micro CT data was compared between different groups at each interval by the Kruskal Wallis test. For each group, Mann–Whitney *U* test was used for comparison between the two time points. The nonparametric method was used to analyze continuous variable data from calvarial defect samples and presented as the mean and standard deviation. A *p* value less than 0.05 was considered statistically significant.

## 3. Results

### 3.1. Clinical and Micro-CT Observations

**Figure 3:**
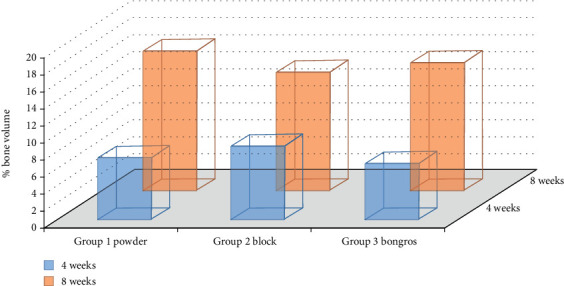
Graphical presentation of percentage bone volume mean values calculated by Micro CT.

**Figure 4:**
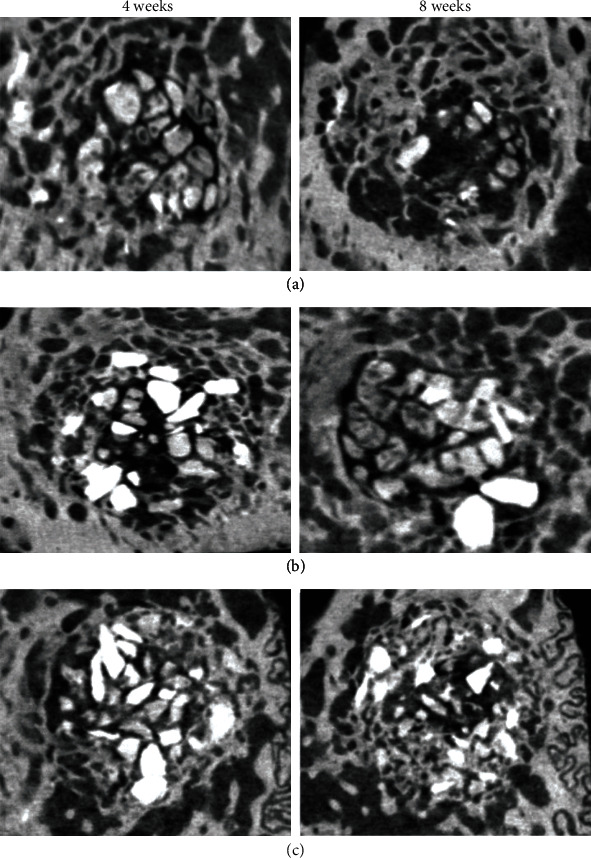
Micro-CT bone volume samples. 3D area of new bone volume formation after 4 weeks. Remaining bone graft at defect site with 3D shadow projection after 8 weeks. (a) Group 1. At 4 weeks, trabecular-shaped bone particles; at 8 weeks, little residual material was observed. (b) Group 2. Block-shaped bone particles were observed after 4 and 8 weeks. (c) Group 3. Graft material was not completely resorbed after 4 and 8 weeks.

All animals remained healthy during the duration of the study without any major complication. The defect sites were free from any clinical sign depicting infection or necrosis. The samples were retrieved after 4 and 8 weeks. The results of the *μ*-CT analysis shown in Figures 3 and 4 are the micro CT bone volume samples at 4 and 8 weeks. We present the values established to characterize the amounts of new bone produced in response to each type of graft material as the average mean bone volumes in Table 1. We calculated the new bone volume (NBV) representing amount of new bone formed and percent bone volume (PBV).

There were statistically significant differences in percentage bone volume (PBV) and new bone volume (NBV) after eight weeks between the powder and block-type graft materials (*p* = 0.01). The PBV in groups 1 and 2 at four weeks after the operation were 7.27% and 8.60%, respectively, and that in the control group was 6.55% (Table 1), and this was a statistically significant difference (*p* = 0.005).

The PBVs in groups 1 and 2 at eight weeks after the operation were 16.33% and 13.92%, respectively, and that in the control group was 14.90%, also a statistically significant difference (*p* = 0.005). There was about 9% increase in PBV after 8 weeks in group 1, and group 2 and 3 showed 5% and 7% increase. The NBV values in groups 1 and 2 at four weeks were 6.45 mm3 and 8.50 mm3, respectively, and that in the control was 7.73 mm^3^.

Comparisons of the bone volume and percentage bone volume in the three bone graft samples showed that percent volume and bone volume were statistically significant at eight weeks for the powder-type bone graft group showed the highest increase in total bone volume, and the block type showed the lowest (Table 1).

### 3.2. Histologic Result

**Figure 5:**
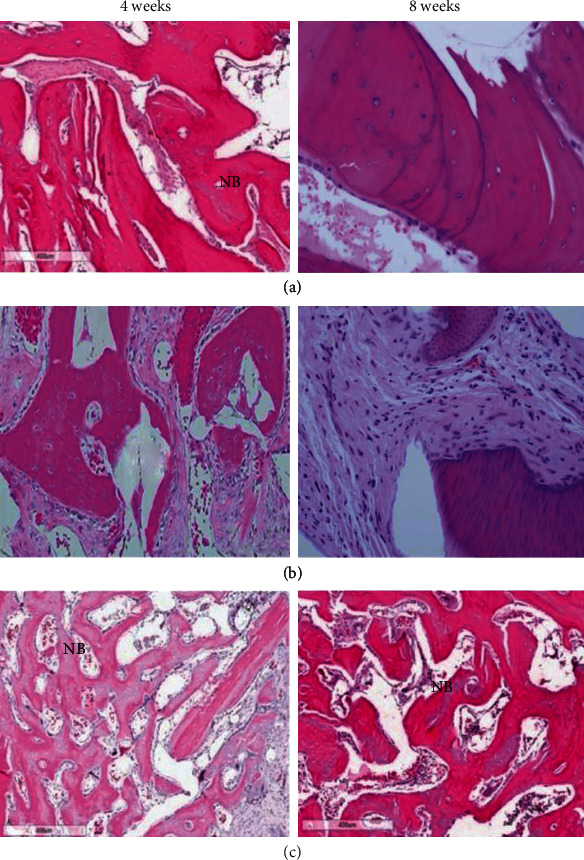
(a) Group 1. Bony bridging as early as 4 weeks. Fibrous connective tissue was observed over scaffold of defect. At 8 weeks, no inflammatory cells. Viable lamellar bone with osteoblast was seen. (b) Group 2. No sign of inflammatory cells. Minimal infiltrated with newly formed bone. At 8 weeks, graft was maintained with no signs of resorption, and osteoid was observed at the periphery. (c) Group 3. At 4 weeks, presence of new bone and blood vessel. Highly lamellated new bone (NB) with rapid growth. Rapid turnover of denser bone after 4 weeks with active resorption. At 8 weeks, new bone formation was increased. (NB: new bone).

Histological analysis of experimental group 1 (Figure 5(a)) shows bony bridging as early as 4 weeks. The newly formed bone and graft materials were in immediate contact, with extensive vascular tissue and highly lamellate new bone growth. At 8 weeks, residual materials were observed on the perimeter, and new bone growth was preserved in the space filled with a matured highly lamellated pattern with residual materials. Group 2 (Figure 5(b)) showed that at 4 weeks, the graft was maintained under the connective tissue layer with no change in the perimeter. There was minimal resorption of graft materials, the morphology was still intact, and the material maintained its shape over the healing period. Block graft was infiltrated by newly formed bone over the residual bone with no signs of severe inflammation, necrosis, or osteolysis. In the control group (Figure 5(c)) at 4 weeks, new bone and blood vessels with highly lamellated new bone and rapid turnover of denser bone showing active resorption were observed. Osteoids at different stages of maturity were observed in the periphery. At 8 weeks, new bone formation was increased. There were areas of active bone healing with rapid turnover of graft and dense bone formation.

## 4. Discussion

Tooth biomaterial constitutes about 55% inorganic and 45% organic substances. Hydroxyapatite (HA) an inorganic substance can combine and dissociate calcium and phosphate, as occurs in bone. BMPs and proteins the organic constituents encompassing osteoinduction capacity, along with type I collagen, like that in the alveolar bone. Tooth biomaterial also reduces the risk of foreign body reactions because of its genetic homogeneity. Similar constituents of tooth biomaterial and bone enhance their bone remodeling capacity as an autogenous bone graft [13]. Auto tooth bone graft particle size can range from powder block or type. The block-type particles can maintain space exhibiting osteoconduction properties, and their blood wettability property helps with osteoinduction. Remodeling occurs in block-type grafts because of space maintenance over a specific time period. The powder type is available in a wide range of particle sizes, different properties of graft materials like porosity between particles, blood wettability, subsequently osteoconduction and osteoinduction, and creeping substitution abilities also varies [11]. Based on the above data, the tooth biomaterial has potential uses in clinical situations as it exhibits excellent bone regeneration by osteoinduction and osteoconduction capacity.

In our study, we found that smaller-sized particles enhance osteogenesis as compared to block-type particles. These findings agree with the results of Pallesen et al. [14] and Kim et al. [15] but disagree with the findings of Kon et al. [16]. Many other studies have also shown the osteogenic superiority of the smaller-sized graft particles [2, 12, 17]; however, the mechanism for particle size affecting the regeneration capacity is not clearly understood. The results in this study indicating more new bone formation associated with small-particle tooth biomaterial than with large particles was observed by histological and micro-CT analysis. Additionally, small-sized particles of tooth biomaterial tend to display a significant amount of resorption than large-sized particles. Small-sized powder-type graft can provide more surface area around which bone may be formed. Thus, particle size is an important factor when comparing the osteogenic potential of the tooth biomaterial. The graft material granules ranging from 75 to 500 *μ*m are considered ideal [17–19]. Smaller particles exhibit low crystalline carbonic apatite which enhances the osteoinduction capacity [20]. An increase in surface area increases the osteoinductive effect and stimulates osteogenesis by increasing pore vascular channel [21]. Implanted composites of human DDM and rhBMP-2 showed better induction of bone formation than observed earlier. Exogenous rhBMP-2 that adsorbs to a fragmentized tooth root matrix exhibits osteoinductivity as autogenic bone [22]. Osteoinductive proteins along with osteoconductive scaffolds show encouraging results combined with osteoconductive proteins, as demonstrated by the bone regeneration induced by rhBMP-2 as a growth differentiation factor delivered on the powder-type demineralized dentin matrix carrier covering significantly more bone area [19].

Despite its promising therapeutic potential, improvements are required for the clinical use of rhBMP-2, particularly its delivery system. Slow degradation of the rhBMP-2 scaffold can result in transplantation of osteoclast progenitor cells, which may cause resorption of the root structure of the adjacent tooth [23]. To overcome these problems, numerous natural and synthetic biomaterial carriers of rhBMP-2 have been examined in the past decade. Biomaterial carriers maintain the concentration of BMPs at the defect site and contribute a structural matrix for bone growth. They may be helpful in providing controlled and sustained release of BMPs to mimicking natural bone healing in vivo [24, 25]. Despite the advancements, an effective carrier of rhBMP-2 is yet to be established [26]. The use of a carrier avoids the need for large amounts of BMP to attain adequate bone induction [27]. Moreover, purified BMP being highly soluble in vivo dislodges instantly following implantation, exerting no effects on bone induction [19]. Based on these findings, BMP requires a suitable carrier for clinical use; thus, experiments using tooth biomaterial with different particle sizes were conducted.

## 5. Conclusions

The results of the study suggest that smaller particles plus rhBMP-2 increase osteogenesis and are preferable to larger particles. We determined the osteoinductivity of tooth biomaterial using histological and micro-CT analysis. There were some limitations to this study. More than 8 weeks of observation is recommended, and the osteogenic activity of grafting materials should be evaluated with better methods to quantify osteogenesis. Histologic evaluation is too subjective for comparative analysis of osteogenic activity. More objective methods for evaluation should be developed for determining the adequate graft particle size in intrabony defects. Osteogenesis quantification to determine of osteogenic activity can be done by more objective method.

## Figures and Tables

**Table 1:** Micro-CT bone analysis result for all groups.

	New bone volume (mm^3^)		*p* value	Percent bone volume (%)		*p* value
	4 weeks (*n* = 6)	8 weeks (*n* = 6)		4 weeks (*n* = 6)	8 weeks (*n* = 6)	
Group 1						
Powder tooth biomaterial + BMP-2	6.45	13.71	0.048	7.27	16.33	0.051
Group 2						
Block tooth biomaterial+BMP-2	8.50	10.40	0.231	8.60	13.92	0.191
Group 3						
Synthetic bone+BMP-2	7.73	10.85	0.138	6.55	14.90	0.125

Data are presented as the mean values of new bone formation.

## Data Availability

The data used to support the findings of this study are available from the corresponding author upon request.
